# Evaluation of the Shelf Life of *Myristica*-*fragrans* Powder-Flavored Oils Obtained through the Application of Two Processes: Infusion and Co-Pressing Technology

**DOI:** 10.3390/molecules29153588

**Published:** 2024-07-30

**Authors:** Irene Maria Grazia Custureri, Monica Rosa Loizzo, Vincenzo Sicari, Roberta Pino, Rosa Tundis, Ana Cristina Soria, Angelo Maria Giuffrè

**Affiliations:** 1 Department of Agraria, University “Mediterranea” of Reggio Calabria, Salita Melissari, Località Feo di Vito, 89124 Reggio Calabria, Italy; irene.custureri@unirc.it (I.M.G.C.); amgiuffre@unirc.it (A.M.G.); 2 Department of Pharmacy, Health and Nutritional Sciences, University of Calabria, Polifunzionale Building, via P. Bucci, 87036 Rende, Italy; robertapino95@gmail.com (R.P.); rosa.tundis@unical.it (R.T.); 3 Institute of General Organic Chemistry (IQOG-CSIC), Juan de la Cierva 3, 28006 Madrid, Spain; acsoria@iqog.csic.es

**Keywords:** mace, *Myristica fragrans*, olive oil quality parameters, shelf-life, obesity, functional food

## Abstract

This work aimed to evaluate the impact of enrichment processing on the quality parameters, bioactivity and sensorial aspects of *Myristica fragrans* (mace)-flavored olive oil storage for one year. The mace powder was added to extra virgin olive oil through two different processes: immediately after crushing the olives by mixing mace (1% weight/weight (*w*/*w*)) with the olive paste (MAVOO-M) and by adding mace to extra virgin olive oil (C) (2% *w*/*w*) (MAVOO-I). A multi-analytical approach was applied to measure the main qualitative indexes, such as the free acidity, peroxide value and ultraviolet parameters. The total phenolic and carotenoid contents (TPC and TCC, respectively) and α-tocopherol were also evaluated, as well as the sensory attributes. The radical scavenging potential was estimated by using two different in vitro tests, namely, 2,2’-azino-bis(3-ethylbenzothiazoline-6-sulfonic acid) (ABTS) and 2,2-diphenyl-1-picrylhydrazyl (DPPH). A significant increase in the free acidity parameter was found in all the flavored oils, and particularly in the MAVOO-M (1.27% oleic acid); at the same time, this oil was the sample with the lowest peroxide value (i.e., 9.68 meqO_2_/kg) after 360 days of storage. At the end of the storage, an increase in L* values was found in both the MAVOO-M and -I vs. the C (43.88 and 43.02, respectively, vs. 42.62). The TCC was strongly influenced by the addition of mace, especially when the infusion process was used. In fact, after one year of storage, the TCC in the MAVOO-I resulted in ~34.7% more than the MAVOO-M. A promising DPPH radical scavenging activity was observed independently by the applied aromatization process, with IC50 values of 19.77 and 17.80 μg/mL for the MAVOO-M and MAVOO-I, respectively. However, this activity decreased during storage, and a similar trend was observed using the ABTS test. In conclusion the infusion as enrichment methodology led to more promising results in terms of functionality compared with the co-mixing one.

## 1. Introduction

Foods are the main source for the maintenance of vital functions in human beings. In addition to providing energy intake, foods are the main source of nutrients that have beneficial effects on human health, for instance, by fighting free radicals through the intake of foods that are naturally rich in anti-scavenging molecules, such as fruit, vegetables and berries [1]. Many studies evaluated the direct activity between food and benefits on human health [2]. In this context, the concept of functional food was born. It was first defined in Japan in 1980s as a food that exerts specific beneficial functions for human health [3]. Foods are linked with diet and play an important role in the prevention of some chronic diseases. Obesity is mainly caused by an excessive calorie intake and a low energy expenditure [4]. It is related to a series of serious chronic pathologies [5]. The most common approaches used to treat this pathology are through laparoscopically mini-invasive surgery, which can cause a weight loss of 70% of excess kilos, or using drugs, such as orlistat or liraglutide, which, however, have undesirable effects on human health [6]. Thus, the best approach to reduce the body fat index, thus significantly improving the reduction and prevention of obesity, remains following an adequate diet with appropriate physical exercises [7]. Another more sustainable way to reduce the predisposition to obesity is using functional foods, clearly while also following a healthy lifestyle. It appears that functional foods are a good approach in treating not only obesity but also the related metabolic syndrome [8,9]. Consumers’ interests are increasingly moving toward healthier foods with beneficial properties for human health. Furthermore, consumers tend to want to reduce the consumption of drugs, preferring the use of natural products acting as “preventives” of some chronic diseases. It is known that herbs, but especially spices, exert beneficial effects on human health and are considered as therapeutic and medicinal foods [10,11]. This potential is due to the complexity of their composition and to the diversity of the mechanisms of action [12].

Extra virgin olive oil is the main fat source of the region of the Mediterranean basin and, together with cereals, legumes, fruits, vegetables, meat and fish, constitutes the Mediterranean diet, which is well known for its countless benefits on human health [13,14]. The Ottobratica is one of the main cultivars growing in the region of Calabria, especially in the Tyrrhenian side of the Reggio Calabria province. The oil derived from this cultivar has been the focus of numerous studies due to its high qualitative characteristics and the interest for this geographical area [15].

In this context, the zest for research in the development of functional foods is increasing and several academics suggested that the addition of vegetable matrices to an olive oil could exert intriguing results in terms of flavor, increased stability or as an alternative to the unflavored olive oil [16,17,18].

*Myristica fragrans* HOUTT is a spice indigenous to Indonesia and is also farmed in Thailand, India and Malaysia [19]. It is characterized by a pleasant smell. The fruit is composed of the seed, representing the nutmeg spice, which is enclosed by the aril that represents the mace spice, and covered by the shell, which represents the flesh. Nutmeg and mace are the main spices derived from this species [20]. Mace is used as a traditional spice in savory dishes or as medicine to treat nausea or dysentery [20]. It exhibits antibacterial, anti-fungal, anti-thrombotic and anti-inflammatory medicinal properties; possesses aphrodisiacal properties, anti-carcinogenic and anti-tumor potential; and is used in menopausal health issues [21,22]. Studies demonstrated how it works as a selective PPARγ modulator that enhances insulin resistance and exhibits anti-obesity effects [23].

New trends have highlighted that consumers today are attentive to new sensory sensations, with particular attention to health and well-being. This has led to the rediscovery of flavored and fortified olive oils, not only through the addition of traditional aromas and flavors but also by adding uncommon flavors and also through the use of new flavoring processes [18,24].

Several enrichment techniques can be used for flavoring olive oils: the addition of bioactive extracts or essential oils, infusion or coextraction [24,25,26,27].

The olive oils thus obtained are oils where the flavoring matrix promotes a set of differentiated sensory characteristics [27,28].

In this study, we provided for the first time a study related to the addition of mace using two different methodologies, which was conducted for one year and periodically analyzed to determine its functionality. Particularly, our work analyzed the bioactivity correlated to obesity and metabolic syndrome through lipase, α-glucosidase and α-amylase enzyme inhibitory assays. Moreover, analyses to evaluate the quality parameters, as well as quantitative parameters and parameters that affect consumers’ acceptability of the AVOOs compared with the control, were also evaluated.

## 2. Results and Discussion

### 2.1. Quality Parameters

The quality parameters of the non-aromatized (control, C) and aromatized olive oils (AVOOs) by co-mixing (MAVOO-M) and by infusion (MAVOO-I) are reported in Table 1. C could be classified as extra virgin olive oil, as established by regulation 1348/2013 of the European Union Commission [29], given its free acidity level of 0.68%; its peroxide value of 9.45 meqO_2_/kg; its extinction coefficients K_232_ and K_268_ of 2.46 and 0.22, respectively; and ΔK value of −0.003. During storage, the free acidity, K_232_ and K_268_ values exceeded the regulatory limits with a constant increase, exceeding the legal limit for extra virgin, whereas the peroxide values (17.89) remained below the maximum limit of 20 meqO_2_/kg of oil. Esposto et al. [30] highlighted the existence of a positive correlation between the increase in the quality parameters and the duration of the storage. In the flavored samples, some parameters exceeded the values established to be classified as extra virgin [27]. In fact, the free acidity value of the MAVOO-I increased from 0.68% to 1.23% between the beginning and the end of the storage period, respectively.

The MAVOO-I showed lower values that ranged between 0.68% (at the beginning of the storage) and 1.23% (at the end of the storage). In both the applied variables, the peroxide value remained below the legal limits during the year of study: from 5.32 to 9.68 meqO_2_/kg of oil in the MAVOO-M and from 9.40 to 17.67 meqO_2_/kg of oil in the MAVOO-I. Worthy of note is the protective effect of the co-mixed mace in this primary oxidation index, and conversely for the other sample. Compared with the extinction coefficients, both the aromatized samples exceeded the values of the control, especially the MAVOO-M. As reported by Díaz-Montaña et al. [31], the increase in free acidity and in K_232_ in the aromatized samples could be due to the presence of free acids in the matrix used for the aromatization. In our samples, a high free acidity value was found at the beginning of storage in the MAVOO-M sample, as opposed to the MAVOO-I, in which the increase was found at the second storage date. These conditions could have been due to the different solubilization times, which took place at the time of infusion in the MAVOO-I sample. In fact, at the end of storage, the two aromatized samples reached very similar values to each other and higher values than the control. The K_268_ coefficient was strongly influenced by the content of a special class of polyphenols: the higher the polyphenol content, the higher the resistance to the secondary oxidation of the oil [30]. The MAVOO-M showed the highest K_268_ value (1.40). These data are in contrast with those found by Diaz-Montaña et al. [31], who aromatized an extra virgin olive oil with 5% basil and rosemary leaves and observed K_268_ values that were lower than the control at T0 and underwent a slight increase from the third month of storage. Conversely, the K_232_ values during storage were higher than the control, in agreement with our results. Fagundes et al. [32] studied a pink-pepper-flavored Brazilian olive oil, in which they found the K_232_ and K_268_ values to be significantly higher than in the control. The authors explained how the presence of some terpenes (of which mace is naturally rich) can interfere with the signal in the 232 nm region, causing an increase in K_232_ [33]. ΔK was strongly influenced by the addition of mace, particularly when it was co-mixed with the olive paste: −0.003 vs. 0.031 for the C and MAVOO-M, respectively, on the day of the production. The coefficient was also influenced by the storage time in the MAVOO-I sample, which reached the value of 0.027 after one year. 

### 2.2. Quantitative Parameters

Carotenoids and chlorophylls are pigments that are naturally present in olive oils. They are responsible for the color of the oil, which can highly vary from greenish to, in some cases, reddish shades. Chlorophylls play an important role in maintaining the oxidative stability of the oil during storage, whereas carotenoids are positively correlated with antioxidant activity thanks to their ability to trap free radicals [34]. Furthermore, chlorophylls and carotenoids are very sensitive to light and oxygen, and their degradation is a complex phenomenon that generates compounds that are not easily identifiable [35]. 

**Figure 1:**
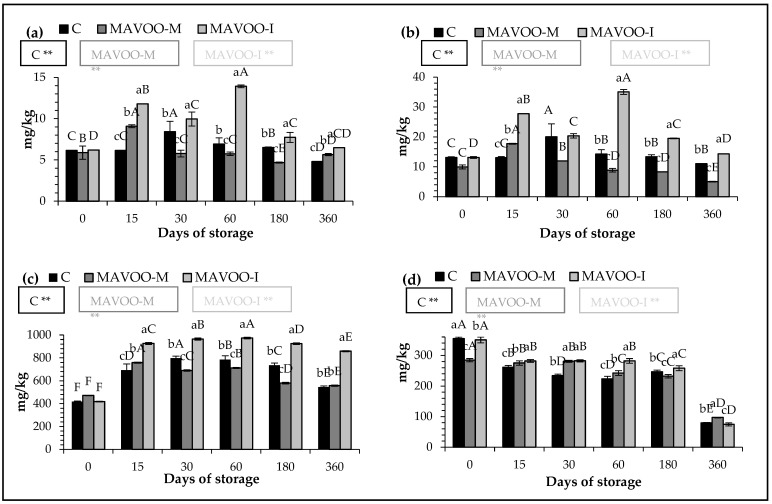
Quantitative parameters of the unaromatized olive oil (C), aromatized olive oil by co-mixing 1% (MAVOO-M) and aromatized olive oil by infusion 2% (MAVOO-I). (**a**) Total carotenoid content (TCC); (**b**) total chlorophyll content (TChlC); (**c**) total phenolic content (TPC); (**d**) α-tocopherol content (α-Toc). Data are expressed as the mean ± standard deviation (*n* = 3). Statistical analysis was followed by Tukey’s test, which were used to evaluate any differences at the same time of analysis (lowercase letters) or during the considered storage (uppercase letters). Results followed by different letters were significant at *p* ≤ 0.01. ** *p* ≤ 0.01.

The total carotenoid content (TCC) (Figure 1a) was strongly influenced by the addition of mace, especially in the sample produced via infusion. In the MAVOO-M, the value of TCC increased from the 15th day after its production. Starting from the 30th day, the content strongly decreased, but maintained a stable value for the rest of the storage, at the end of which, it stabilized at values higher than the control (5.64 vs. 4.8 mg/kg). In the MAVOO-I sample, the TCC showed significantly higher values during the entire storage period compared with the other samples (*p* < 0.01). After one year, it maintained a value of 6.47, which was about 34.7% more than the MAVOO-M sample and 14.7% more than the C sample. Loizzo et al. [36] enriched an extra virgin olive oil with different varieties of Capsicum annuum and Capsicum chinese and found that only the variety of C. chinese “red mushroom” showed a notable increase in the TCC content (18.4 vs. 28.8 mg/kg). The rest showed an increase of less than 40%, as in our study.

The content of total chlorophylls (TChlC) (Figure 1b) was also strongly influenced by the addition of mace (*p* < 0.01). Lower and very unstable values were observable when analyzing the data from the MAVOO-M sample. It maintained values even lower than the control for the entire conservation period (from 13.09 vs. 13.12 to 11.03 vs. 4.05 mg/kg). The data from the MAVOO-I sample showed how up to the 60th day of storage, this content increased until reaching the maximum values of 35.04 mg/kg. Through enrichment with rosemary leaves, the authors observed values for flavored oils that were 2.5 times higher than the oil on its own. Considering the 60th day from production as the comparison time, that is to say, the moment of maximum value in the MAVOO-I sample, the increase corresponded to 2.45. This finding agrees with our data [37].

Esposto et al. [27], by analyzing 14 different cultivars of extra virgin olive oil, showed how the total phenolic content (TPC) is a highly variable factor by finding values between 18 and 1476 mg/kg. Our C sample exhibited 418.51 mg/kg (Figure 1c). At the beginning of the storage, the MAVOO-M had the highest TPC value compared with the MAVOO-I (471.51 vs. 418.54 mg/kg). As the storage progressed, the spice in the infusion dissolved and showed promising levels that were maintained throughout storage, where they reached up to 887.59 mg/kg over time. The MAVOO-I had values that were 1.56 times higher than the C and 1.53 times higher than the MAVOO-M. Issaoui et al. [37] did an enrichment with lemon, onion, garlic and paprika and saw how the polyphenol content was highly influenced by the spice (lemon 467.5, onion 505.47, garlic 427.8 and paprika 461.3 mg/kg). They also demonstrated how, by subjecting these samples to accelerated conditions of 60 °C for 8 h, in the onion-flavored sample, the TPC value reached 597.9 mg/kg, whereas in the garlic-flavored sample, it reached 490 mg/kg after only 1 h. The other samples, if exposed to accelerated conditions, suffered a decrease in TPC. Therefore, as can be extrapolated from various studies, the phenolic content is strongly influenced by the ingredient used for the aromatization [37,38].

α-Tocopherol is an important molecule present in nature, of which olive oil is the main nutraceutical source. Recent studies have reported its beneficial properties for human health, but also its effects on maintaining the shelf-life of the olive oil. Moreover, links with antioxidant activity were also found [15]. Our unflavored sample had a medium–high α-tocopherol level of 354.63 mg/kg (Figure 1d). Sicari et al. [15] observed values between 100.15 mg/kg (Nocellara del Belice cultivar) and 175.15 mg/kg (Ottobratica cultivar). This parameter is generally strictly varietal and depends on the cultivation year, as well as on the cultivar. Another factor that negatively affects the content of this molecule is the extraction method. In fact, it seems that the three-phase extraction system, which involves the use of water to increase the oil extraction yield, causes a strong decrease in α-tocopherol [15]. Regarding our flavored samples, the values of the MAVOO-M and MAVOO-I corresponded to 284.6 and 350.6 mg/kg, respectively. Despite this initial condition, the oil produced by co-mixing at the end of the storage was much more stable and richer than that produced by infusion, which showed accentuated degradation by exhibiting values of α-tocopherol that were even lower than the control (Figure 1d) (97.21 and 74.78 vs. 79.53 mg/kg for the MAVOO-M, MAVOO-I and C samples, respectively). Some unexplained behaviors were observed for these quantitative parameters, especially for the MAVOO-M sample, during the considered storage period. Studies have reported how some chemical and/or enzymatic reactions due to the greater exposure of the olive paste to oxygen or light could induce the activity of the lipoxygenase (LOX) complex and, therefore, the development of this “anomalous” data [32].

### 2.3. Parameters that Affect Consumers’ Acceptability

As can be seen from Table 2, the color was significantly influenced by the addition of mace (*p* < 0.01). As reported by Sikorsa et al. [36], color changes in an olive oil are part of the natural preservation processess and can also start from the first month of storage. From the data obtained, an increase in the lightness (L*) values could be observed in all the samples. An exception was the MAVOO-M sample, in which this increase was delayed and took place starting from the sixth month of storage. In the other samples, the increase started during the first months of storage, in accordance with Sikorsa et al. [39]. This confirmed the photo-oxidative protective effects of the compounds derived from mace, which, however, did not exert the same effect in the sample produced by infusion. Significant variations (*p* < 0.01) were found in the parameters for the analysis of the red–green (a*) and yellow–blue (b*) shades. It is worth pointing out how the two AVOO samples reacted differently. In fact, in the MAVOO-M, from the day of its production until the end of its conservation, there was a variation between 3.44 and 0.17 in the a* value, whereas in the MAVOO-I sample, the values were between 3.42 and −0.01. Instead, the b* values varied by 187 and 155% in the MAVOO-M and MAVOO-I, respectively. Chroma* offers a numerical evaluation of the color intensity [39]. Observing this parameter, the brightest sample appeared to be the MAVOO-M, with a value at T0 of 6.85 vs. 7.21 and 7.23 for the MAVOO-I and C, respectively. In all the samples, both unflavored and flavored, decreases were recorded in the Chroma* values. Particularly in the MAVOO-M, this decrease was more pronounced than the others (69.78% vs. 65.42 and 69.02% for the MAVOO-I and C, respectively). 

**Figure 2:**
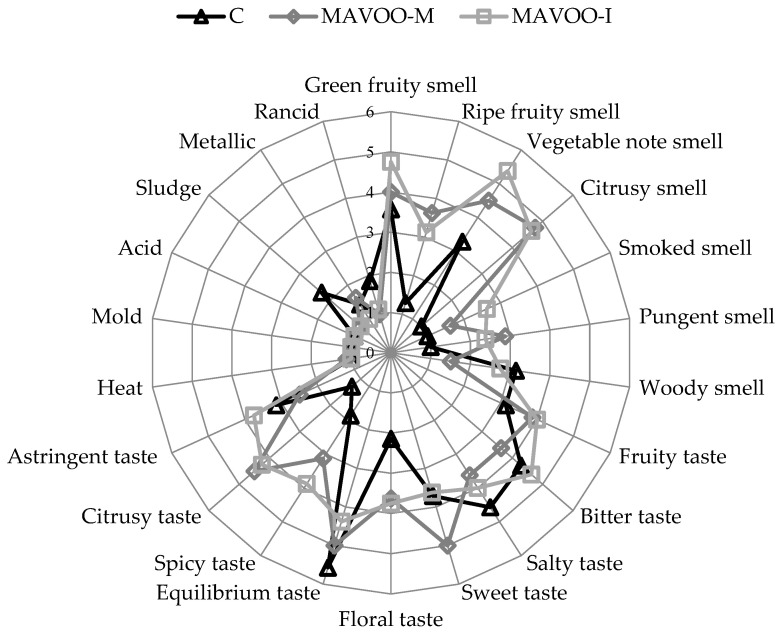
Sensory attributes of the unaromatized olive oil (C), aromatized olive oil by co-mixing 1% (MAVOO-M) and aromatized olive oil by infusion 2% (MAVOO-I).

Figure 2 shows the sensory attributes of the control and aromatized oils at T0. The tasters strongly appreciated the product, with equal values between the two enrichment methods used. None of judges were able to correctly define the enrichment matrix, mistaking it simply for “nutmeg”. The C had a slight defect, which was identified by a “sludge” note. In the two different aromatized oils, this defect was perfectly masked. Unfortunately, in the MAVOO-M sample, a new defect defined by a “metallic” note was highlighted. This could have been due to the combination of the mace volatiles with those of the olive oil. Regarding the olfactory characteristics, a new “smoked” note was found in the aromatized samples, particularly in the MAVOO-I sample, which was also characterized by the highest “vegetal” and “green-fruity” notes. A new “citrusy” note appeared in the AVOOs, which is typical of mace. Regarding the taste component, a “bitter” note was the predominant in the MAVOO-I sample, whereas a “sweet” note dominated in the MAVOO-M sample. Additionally, from the taste analysis, a “citrusy” note emerged, which was perceived slightly more in the MAVOO-M sample than in the MAVOO-I. In summary, both samples received positive ratings from the expert panelists.

### 2.4. Antioxidant Activities

**Figure 3:**
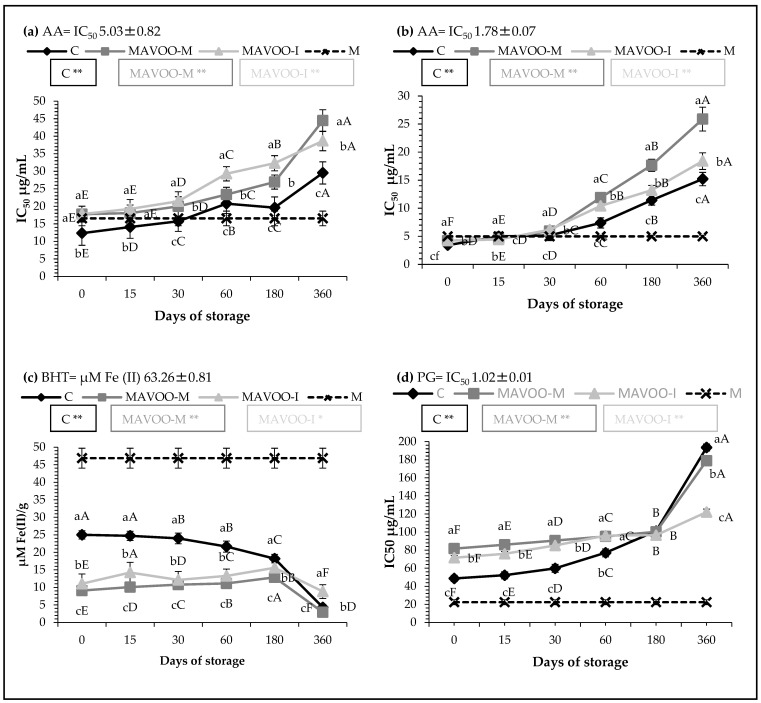
Antioxidant and antiradical activities of the unaromatized olive oil (C), aromatized olive oil by co-mixing 1% (MAVOO-M) and aromatized olive oil by infusion 2% (MAVOO-I). (**a**) DPPH test (AA: ascorbic acid positive control); (**b**) ABTS test (AA: ascorbic acid positive control); (**c**) FRAP test (BHT: butylated hydroxytoluene positive control); (**d**) β-carotene bleaching test (PG: propyl gallate positive control). Data are expressed as the mean ± standard deviation (*n* = 3). Statistical analysis was followed by Tukey’s test, which were used to evaluate any differences at the same time of analysis (lowercase letters) or during the considered storage (uppercase letters). Results followed by letters were significant at *p* ≤ 0.01. * *p* ≤ 0.05; ** *p* ≤ 0.01.

Antioxidant molecules provide a valid contribution to the management of oxidative stress, which is associated with various chronic diseases. Moreover, antioxidant activity can influence the shelf-life of a product over time. Studies showed that antioxidant activity is the result of a complex mechanism of chemical reactions involved in a series of different processes. In this context, it is recommended to have an overall view of this activity in order to have a multi-analytical approach [40]. Additionally, antioxidant activity is closely correlated with the content of polyphenols [20,40]. In this research, the antioxidant capacity of these aromatized oils was tested using various in vitro assays, including the “scavenging” of free radicals through the DPPH radical and the ABTS radical cation, which have a different stereochemistry and a different mechanism of action. Nevertheless, both detected the chain breaking potential of the tested extracts by measuring the transfer of hydrogen to free radicals [38]. The dry extract of mace (M) exhibited IC_50_ values of 16.56 and 4.99 μg/mL in the DPPH and ABTS tests, respectively (Figure 3a and 3b). Li et al. [41] detected IC_50_ values of 39.65 and 27.68 μg/mL in an ethanolic extract of nutmeg in DPPH and ABTS tests, respectively, where they identified ethanol and methanol as the best solvents in the extraction of the antioxidant compounds from this matrix. Loizzo et al. [20] found very similar IC_50_ values to Li et al. [41], with 39.6 and 32.7 μg/mL for the mace extract in the DPPH and ABTS tests, respectively. The extracts of our aromatized oils showed promising IC_50_ values of 17.77 and 17.80 μg/mL in the DPPH test for the MAVOO-M and MAVOO-I, respectively (Figure 3a). These values were comparable with the values of the C sample, with an IC_50_ of 12.33 μg/mL. As the storage period progressed, there was a natural increase in these values and a progressive decrease in the radical scavenging potential until reaching IC_50_ values of 29.54, 44.45 and 38.66 μg/mL for the C, MAVOO-M and MAVOO-I, respectively, after one year of storage. This increase was greater in the flavored oils compared with the control, which is a sign that the bioactive compounds of the mace responsible for the scavenging activity that were qualitatively detectable through the DPPH assay were less stable over time compared with those of the olive oil. In particular, the increase started from the sixth month of storage and the MAVOO-M and MAVOO-I reached values that were 50.4% and 30.9% higher than the C, respectively.

By analyzing the data that resulted from the ABTS test (Figure 3b), an analog situation could be observed. The MAVOO-M and MAVOO-I presented IC_50_ values at the day of production of 4.21 and 4.23, respectively, vs. 3.43 μg/mL for the C. In this case, after one year of storage, an exponential increase was more observable in the sample produced through co-mixing (IC_50_ 25.89 μg/mL) rather than in the sample produced by infusion, which reached comparable values with the control (IC_50_ 15.21 vs. 18.4 μg/mL for the C and MAVOO-I, respectively). 

The ability of the sample to induce the reduction of the ferric complex (Fe^3+^) to a ferrous complex (Fe^2+^) by stabilizing it was measured through the FRAP test. Furthermore, conversely to the two assays previously discussed, through this test, a qualitative evaluation regarding the transfer of electrons from the antioxidant to the metal ions was carried out [39]. The FRAP values obtained from the M sample corresponded to 46.88 μM Fe(II)/g, which was slightly lower than the positive control (63.2 μM Fe(II)/g) (Figure 3c). Loizzo et al. [20], by analyzing mace, also found FRAP values only slightly higher than BHT and equal to 68.7 μM Fe(II)/g. Instead, Trifan et al. [42], when studying the essential oil of *Myristica fragrans* H., found FRAP values equal to 105.28 mg TE/g. Our control oil also showed values below BHT (25.01 μM Fe(II)/g), and during the storage, it completely lost its low initial activity (4.31 μM Fe(II)/g). Therefore, the samples obtained from the union of two poorly active products were obviously characterized by a poorly or even non-existent reducing power activity (Figure 3c). Custureri et al. [43], by adding ginger dried powder to the olive paste, obtained promising FRAP values (86.42 μM Fe(II)/g), despite the non-aromatized olive oil possessing poor reducing power. Similarly, Loizzo et al. [36], by flavoring an olive oil with chilli pepper, found that all values were higher than the positive control BHT and between 129.8 and 139.5 μM Fe(II)/g. This means that the matrix plays a fundamental role in transferring this potential to the aromatized oils. 

Figure 3d graphically represents the values regarding the inhibition of lipid peroxidation evaluated through the β-carotene bleaching test, in which β-carotene acted with the radicals resulting from the oxidation of an emulsion containing linoleic acid. The values obtained for the MAVOO-M and MAVOO-I samples were approximately 1.7 and 1.5 greater than the C, respectively, and during storage, these values increased exponentially. The C had poor activity, with an IC_50_ value of 48.72 μg/mL. Despite this, studies confirmed that this activity is not very scarce. In fact, Plastina et al. [44] found IC_50_ values for the Roggianella and Dolce di Rossano cultivars of 127 and 205 μg/mL, respectively, which were approximately 2.6 and 4.2 times lower than our C sample. 

Conversely, the M sample showed a promising value of 22.39 μg/mL. The data obtained from the aromatized samples show that these offered no increased protection against lipid peroxidation compared with the natural protection of the non-aromatized oil.

### 2.5. Carbohydrate-Hydrolyzing Enzymes and Lipase-Inhibition Activities

Samples were also tested in terms of the inhibitory capacity toward enzymes involved in carbohydrate dygestion, such as α-amylase and α-glucosidase (Table 3). The hypolipidemic activity was instead evaluated through the inhibition of pancreatic lipase. This enzyme is involved in fat metabolism and its inhibition determines better control of the lipid profile in the human body [45]. The M sample in the inhibitory activity assay against the α-amylase enzyme presented values that were clearly higher than the acarbose used as a positive control (IC_50_ 162.49 vs. 50.18 μg/mL) (Table 3). The authors demonstrated how mace has a powerful effect on these enzymes and how it can be used in the formulation of drugs for the treatment of diabetes mellitus [46]. Other researchers explained how some terpenes, such as α- and β-pinene, myristicin or sabinene, of which mace is naturally rich, are potent anti-diabetic agents [41]. Sivaraj et al. [46], when analyzing the bioactivity of *Myristica fragrans*, underlined how the inhibition activity of the enzymes was dose dependent. At a concentration of 500 μg/mL, the extract exhibited a potential of 81.3% compared with 98.15% of acarbose used as the positive control. Among the aromatized samples, interesting values were obtained from the MAVOO-I sample, which exhibited, both at the beginning and at the end of the storage, lower values than the C sample (IC_50_ 189.47 vs. 269.02 and 258.65 vs. 289.32 μg/mL for the MAVOO-I and C samples, respectively).

Concerning the inhibitor effects against the α-glucosidase enzyme, the M showed IC_50_ values of 206.17 μg/mL. This lower value was in contrast with those found by Loizzo et al. [20], who, for the same extract, found the promising IC_50_ value of 75.7 μg/mL. The C sample possessed a value (IC_50_ value of 137.34 μg/mL) even lower than the M sample, but with time, lost most of its potential and reached a value of IC_50_ 778.23 μg/mL. Loizzo et al. [47], when analyzing a group of eight different samples of virgin olive oils from the region of Campania, found IC_50_ values between 184 and 766 μg/mL. Moreover, they highlighted how the greater inhibitory activity of these tested oils was found mainly against α-glucosidase rather than against α-amylase. This scientific evidence was completely in agreement with our data. The MAVOO-M and MAVOO-I initially showed values very close to those of the control (IC_50_ values of 136.58 and 136.55 μg/mL, respectively). Differently from sample C, they maintained this inhibitory activity throughout the storage, with values 125.6% and 153.4% lower for the MAVOO-M and MAVOO-I, respectively. 

The bioactive molecules present in mace were shown to have anti-obesity properties. Thus, Vangoori et al. [48] conducted a study on albino mice to observe the effect of mace on food intake and weight managment for 35 days. The results showed that its use decreased food intake, which inhibited hunger and body weight, thanks to its inhibitory activity against pancreatic lipase. With this background, our samples were also tested to evaluate the inhibitor potential on pancreatic lipase enzyme (Table 3). The M presented values higher than Orlistat, which was used as a positive control, by about 2.23 times (IC_50_ 83.6 vs. 37.44 μg/mL). The aromatized olive oil extracts presented promising values at the day of their production of IC_50_ 62.25 and 62.33 μg/mL for MAVOO-M and MAVOO-I, respectively. After one year, MAVOO-I maintained an excellent value of IC_50_ 138.66 μg/mL against the IC_50_ 312.97 μg/mL of the C sample, which was approximately 2.25 times lower. This data confirmed the inhibitory power of mace on the activity of the pancreatic lipase enzyme, which was already studied by other authors, while also giving us positive feedback on its employment in the formulation of functional products, as it maintains its properties and potential.

## 3. Materials and Methods

### 3.1. Preparing the Samples (C, MAVOO-M and MAVOO-I)

Olive oil was derived from Ottobratica cultivar olives (*Olea europea* L.) that were cultivated in orchards in the province of Reggio Calabria in the south of Italy during crop season 2021. A mini-pressing apparatus was used for the oil extraction at the laboratory scale. It was composed of a hammer crusher, a malaxator and a press. After the extraction, it was necessary to separate the olive oil from wastewater, and it was finally saved in dark glass bottles (100 mL), with a headspace between 2 and 5% at ambient temperature and without light. 

The arils of *Myristica fragrans* H. were acquired from an online website in September 2021. It was decided to purchase the arils whole and not in powder form due to its frequent mixing with by-products or other species. The arils were packaged in bags of 100 g each. On the bag label, Sri Lanka was indicated as the country of production and Belgium as the country of packaging. The shelf-life was indicated as three years from the packaging date. They were also classified as products “that have zero or minimal quantities of pesticides or chemical fertilizers, support animal welfare and standards for non-genetically modified animals” through the “Eu Organic” certification. After the production of the control (olive oil as it is, C sample), the arils were ground into a fine powder to increase the contact surface and to increase the bioavailability of the biomolecules in the resulting aromatized olive oils (AVOOs) [16]. There is a great variability in aromatization processes (including percentages), and they are mainly influenced by the type of matrix used [16,17,18]. Thus, after careful bibliographic research and preliminary tests, 2% was chosen. Afterward, the C was infused with 2% of mace spice in relation to the volume of the C for one month in the dark and under permanent shaking. The mace-aromatized virgin olive oil by infusion (MAVOO-I) was thus obtained after a precise filtering step to eliminate any residues of the spice.

With the aim to optimize the production of that type of aromatized olive oil, a second methodology was applied. In this case, after the milling of the olives, an exact quantity of mace powder was weighed (1% of the olive paste) and immediately added to it before mixing in the malaxation phase. The obtained malaxed paste was immediately pressed and filtered to prevent increased contact with oxygen or light, and thus, triggering any oxidative processes. The mace-aromatized virgin olive oil by mixing (MAVOO-M) was thus obtained after a precise filtering step to eliminate any residues of the wastewater.

Knowing the nutritional properties of mace [19,20,21,22,23] and being aware of the maximum period of conservation of an olive oil, which maintains all its properties relating to human health for a maximum of 18 months in very exceptional cases, and generally for 12 months, a precise working plan of analysis was drafted. Six samplings (on the day of production, 15 days after production, 1 month after production, 2 months after production, 6 months after production and one year after production) were planned to evaluate the impact of the evolution of the natural oxidative processes and to estimate whether the enrichment could enhance the stability over time of the olive oil. Moreover, thanks to the countless properties of the spice, in vitro assays were also conducted regarding the antioxidant and the inhibitor enzymatic activity of all the samples produced.

### 3.2. Mace Extract

The whole aril was ground into a fine powder, and the extract was prepared following the method as previously reported by Loizzo et al. [20]. The obtained extract (M) was filtered and stored at 4 °C in the dark until use. 

### 3.3. Quality Parameters of the Samples (C, MAVOO-M and MAVOO-I)

The quality parameters were determined according to the EEC Regulation [29], such as the free acidity (expressed as % oleic acid), peroxide values (expressed as meqO_2_/kg of oil), K_232_, K_268_ and ΔK. 

### 3.4. Pigments Quantitative Determination 

Pigments were extracted using an equal quantity of oil and n-*hexane*. Total contents of chlorophylls (TChlC) and carotenoids (TCC) were determined spectrophotometrically (λ = 670 and 470 nm, respectively) and expressed as mg/kg of pheophytin and lutein, respectively [49]. For the extraction of the phenolics, the method previously described by Montedoro et al. [50] was applied. The oil was mixed with *methanol* (70%) and n-*hexane.* This mixture was centrifuged, and the upper phase was collected, filtered and stored at −20 °C until analysis. 

### 3.5. Total Phenols and α-Tocopherol Contents

The determination of the total phenols content (TPC) of the AVOOs and C was determined following using the methodology of Baiano et al. [51]. The TPC was determined at 750 nm and expressed as mg GAE/kg of oil. 

For the quantification of the α-tocopherol content (α-Toc), a UHPLC-DAD system was utilized following the method of Custureri et al. [43]. The detector was set to an excitation wavelength of 290 nm and an emission wavelength of 330 nm. The identification and quantification were performed by a calibration curve using pure α-tocopherol. The results were expressed as mg/kg of oil. 

### 3.6. Parameters that Affect Consumer’s Acceptability

The colorimetric parameter values were measured with a colorimeter (Konica Minolta CM-700d, Osaka, Japan) according to the international standard CIELab L*, a* and b*. The results were reported as Chroma*.

The C, MAVOO-M and MAVOO-I were judged by a certified organization of experts. The panel was comprised of seven specialist examiners from 30 to 65 years old. The evaluation was done using 9-point scales, where 1 was absent and 9 was extremely perceptible, and some new notes were added for the AVOOs. Quantitative descriptive analysis (QDA) was done to report the sensory attributes of the sample, and the results were drafted as a spider graph. The sensory evaluations were done in accordance with the current legislation and according to the internal regulations of the department. All the panelists were previously informed about the ingredients they tasted.

### 3.7. Evaluation of Antioxidant Activities

Multi-analytical assays were applied to better appraise the real antioxidant or anti-scavenging potential of the samples. The dried extract was used for these determinations.

The 1,1-diphenyl-2-picrylhydrazyl (DPPH) and the 2,2-azino-bis (3-ethylbenzothiazoline-6-sulfonic acid) (ABTS) radical activities were performed as previously reported [50]. Briefly, a solution of DPPH (1.0 × 10^−4^ M) was mixed with the sample (at concentrations in the range of 1–1000 µg/mL). The absorbance was read at 517 nm. For the ABTS assay, a solution of ABTS was prepared and left in the dark for 12 h. A mixture sample (at concentrations in the range of 1–400 µg/mL) and diluted ABTS solution were formulated, and after 6 min, the absorbance was measured at 734 nm. Ascorbic acid was used as the positive control in both the radical scavenging assays. The ferric reducing antioxidant power (FRAP) was executed as previously reported [52]. The FRAP reagent was prepared by mixing 10 mM tripyridyltriazine (TPTZ) solution with HCl, acetate buffer (pH 3.6) and 20 mM FeCl_3_. A mixture extract (2.5 mg/mL), water and FRAP reagent were prepared and incubated for 30 min at 25 °C. The absorbance was measured at 595 nm. The value was expressed as μM Fe(II)/g. Butylated hydroxytoluene (BHT) was used as a positive control. The protection of lipid peroxidation was tested by a *β*-carotene bleaching assay [50]. An emulsion of *β*-carotene, Tween 20 and linoleic acid was mixed with the sample (at a concentration in the range of 5–100 μg/mL). The absorbance was read at λ = 470 nm after 30 min of incubation (at 45 °C). Propyl gallate was used as the positive control.

### 3.8. Evaluation of α-Amylase-, α-Glucosidase- and Lipase-Inhibition Activities

For the inhibition of α-amylase and α-glucosidase enzymes, the method of Formoso et al. [52] was applied. In the α-amylase inhibitory assay, a starch solution of enzyme (EC 3.2.1.1) and colorimetric reagent were prepared. Both the control and extract were added to the starch solution and left to react with the enzyme. The absorbance was read at 540 nm. In the α-glucosidase inhibitory activity test, a maltose solution, enzyme (EC 3.2.1.20) solution and *O*-dianisidine solution were prepared and mixed. This mixture was left to incubate at 37 °C for 30 min. Then, perchloric acid was added. The supernatant was collected and mixed with DIAN and PGO, and was left to incubate at 37 °C for 30 min. The absorbance was read at 500 nm, and acarbose was used as a positive control in both tests. 

For the inhibition of the pancreatic lipase enzyme, the method previously described by Formoso et al. [52] was applied. In this assay, a mixture of samples, 4-nitrophenyl octanoate (NPC), Tris-HCl buffer (pH 8.5) and enzyme solution were added in a 96-well plate and incubated at 37 °C for 30 min. The absorbance was determined at λ= 412 nm and Orlistat was used as the positive control. 

These results were expressed as the 50% inhibitory concentration (IC_50_).

### 3.9. Statistical Analysis

The samples were analyzed in triplicate. The results were expressed as the mean ± standard deviation (S.D.) (*n* = 3). Tukey’s test at *p* < 0.01 was applied to the data using a one-way analysis of variance (ANOVA) by IBM SPSS 21.0 (SPSS Inc., Chicago, IL, USA). ** *p* < 0.01 and * *p* < 0.05 were statistically significant; ns, not significant at *p* > 0.05.

## 4. Conclusions

The addition of this little-known spice with its innumerable nutritional properties and strong sensory characteristics but also toxic effects not only enhanced the flavor of the oil and mitigated some initial defects but also gave an added nutritional value with positive impacts on health, thus generating products that could be defined as functional. Despite this, worthy of note are the quality parameters in which both enrichment technologies led to negative effects. In fact, there was an important increase, which was almost similar between the MAVOO-M and MAVOO-I, in the free acidity and in the extinction coefficients values during the storage compared with the control. The infusion as an enrichment methodology led to more promising results, not only in terms of functionality but also in terms of quantitative parameters, i.e., maintaining the highest values in TCC, TChlC and TPC, even after the entire storage period, compared with the co-mixing one. Thanks to its hypoglycemic effect due to its considerable inhibitory activity against the α-amylase and α-glucosidase enzymes and thanks to its promising activity against the pancreatic lipase enzyme, its extract could be used in formulations thanks to its healthy effects in the treatment of obesity and related pathologies. Hence, our samples could be considered functional but, regrettably, in vivo studies are necessary to confirm its functionality on the human body. Nonetheless, due to the presence of toxic compounds, its use may not be suitable for special groups of people (i.e., pregnant women, children, etc.).

## Figures and Tables

**Table 1:** Quality parameters of the unaromatized olive oil (C), aromatized olive oil by co-mixing 1% (MAVOO-M) and aromatized olive oil by infusion 2% (MAVOO-I).

Sample	Days of Storage						
	T0	T15	T30	T60	T180	T360	Sign
Free Acidity (% Oleic Acid)							
C	0.68 ± 0.02 ^bC^	0.70 ± 0.00 ^bB^	0.71 ± 0.00 ^bF^	0.56 ± 0.00 ^cD^	0.53 ± 0.05 ^cE^	0.84 ± 0.01 ^bA^	**
MAVOO-M	0.93 ± 0.04 ^aD^	0.96 ± 0.00 ^bE^	0.93 ± 0.00 ^bD^	1.06 ± 0.04 ^aB^	0.98 ± 0.01 ^aC^	1.27 ± 0.04 ^aA^	**
MAVOO-I	0.68 ± 0.03 ^bD^	0.98 ± 0.01 ^aB^	0.93 ± 0.03 ^bC^	0.97 ± 0.04 ^bB^	0.92 ± 0.01 ^bC^	1.23 ± 0.02 ^abA^	**
Sign	**	**	**	**	**	**	
Peroxide Value (meqO_2_/kg)							
C	9.45 ± 0.20 ^aD^	9.50 ± 0.36 ^aD^	10.56 ± 0.25 ^bC^	10.95 ± 0.03 ^aC^	12.86 ± 0.09 ^aB^	17.89 ± 0.09 ^aA^	**
MAVOO-M	5.32 ± 0.06 ^bDE^	5.53 ± 0.06 ^cD^	5.81 ± 0.18 ^cC^	6.22 ± 0.04 ^bE^	7.44 ± 0.06 ^cB^	9.68 ± 0.19 ^bA^	**
MAVOO-I	9.40 ± 0.18 ^aE^	8.26 ± 0.11 ^bF^	11.18 ± 0.50 ^aBC^	10.65 ± 0.59 ^abD^	11.83 ± 0.09 ^bB^	17.67 ± 0.45 ^abA^	**
Sign	**	**	**	**	**	**	
K_232_							
C	2.46 ± 0.06 ^cC^	2.47 ± 0.05 ^cC^	1.98 ± 0.05 ^cE^	2.11 ± 0.03 ^cD^	2.87 ± 0.08 ^B^	2.95 ± 0.14 ^A^	**
MAVOO-M	3.80 ± 0.02 ^aA^	2.87 ± 0.02 ^bD^	3.17 ± 0.01 ^aB^	3.08 ± 0.20 ^aBC^	3.00 ± 0.09 ^C^	3.19 ± 0.29 ^B^	**
MAVOO-I	2.57 ± 0.25 ^bD^	3.59 ± 0.18 ^aA^	2.58 ± 0.27 ^bD^	2.59 ± 0.43 ^bD^	2.83 ± 0.10 ^C^	3.44 ± 0.38 ^B^	**
Sign	**	**	**	**	ns	ns	
K_268_							
C	0.22 ± 0.02 ^cC^	0.24 ± 0.02 ^cB^	0.20 ± 0.02 ^cD^	0.20 ± 0.05 ^cD^	0.28 ± 0.01 ^cA^	0.28 ± 0.00 ^cA^	*
MAVOO-M	1.40 ± 0.02 ^aA^	1.14 ± 0.10 ^aD^	0.95 ± 0.02 ^aE^	1.25 ± 0.13 ^aB^	1.29 ± 0.04 ^aBC^	1.21 ± 0.01 ^aC^	**
MAVOO-I	0.28 ± 0.02 ^bE^	0.63 ± 0.01 ^bD^	0.64 ± 0.08 ^bD^	0.66 ± 0.03 ^bC^	1.10 ± 0.01 ^bA^	0.75 ± 0.01 ^bB^	**
Sign	**	**	**	**	**	**	
			ΔK				
C	-0.003 ± 0.000 ^cBC^	-0.003 ± 0.000 ^cC^	-0.003 ± 0.000 ^BC^	-0.003 ± 0.000 ^cBC^	-0.001 ± 0.000 ^cAB^	0.000 ± 0.000 ^cA^	**
MAVOO-M	0.031 ± 0.003 ^aD^	0.035 ± 0.002 ^aC^	0.039 ± 0.002 ^B^	0.043 ± 0.001 ^aA^	0.034 ± 0.003 ^aC^	0.038 ± 0.004 ^aB^	*
MAVOO-I	0.000 ± 0.000 ^bD^	0.012 ± 0.001 ^bC^	0.015 ± 0.002 ^B^	0.015 ± 0.004 ^bB^	0.026 ± 0.002 ^bA^	0.027 ± 0.001 ^bA^	**
Sign	**	**	ns	**	**	**	

Data are expressed as the mean ± standard deviation (*n* = 3). Statistical analysis were followed by Tukey’s test, which were used to evaluate any differences at the same time of analysis (lowercase letters) or during the considered storage (uppercase letters). Results followed by letters were significant at *p* ≤ 0.01. * *p* ≤ 0.05; ** *p* ≤ 0.01; ns, not significant at *p* > 0.05.

**Table 2:** Colorimetric parameter values of the unaromatized olive oil (C), aromatized olive oil by co-mixing 1% (MAVOO-M) and aromatized olive oil by infusion 2% (MAVOO-I).

Samples	Days of Storage						
	T0	T15	T30	T60	T180	T360	Sign
L*							
C	32.70 ± 0.02 ^bD^	32.73 ± 0.07 ^aD^	41.42 ± 0.77 ^aC^	41.96 ± 0.05 ^aBC^	42.08 ± 0.04 ^bB^	42.62 ± 0.01 ^A^	**
MAVOO-M	32.81 ± 0.06 ^aC^	32.24 ± 0.12 ^bD^	32.15 ± 0.06 ^bE^	32.25 ± 0.02 ^bD^	42.84 ± 0.04 ^aB^	43.88 ± 1.16 ^A^	**
MAVOO-I	32.70 ± 0.01 ^bD^	32.17 ± 0.06 ^cE^	42.21 ± 0.06 ^abB^	41.73 ± 0.03 ^abC^	42.12 ± 0.01 ^bBC^	43.02 ± 0.06 ^A^	**
Sign	*	**	**	**	**	ns	
a*							
C	3.42 ± 0.02 ^A^	3.43 ± 0.03 ^A^	0.05 ± 0.03 ^bD^	0.73 ± 0.01 ^bB^	0.15 ± 0.01 ^aC^	−0.06 ± 0.01 ^cE^	**
MAVOO-M	3.44 ± 0.02 ^B^	4.21 ± 1.17 ^A^	3.32 ± 0.02 ^aC^	3.34 ± 0.01 ^aC^	-0.01 ± 0.01 ^bE^	0.17 ± 0.02 ^aD^	**
MAVOO-I	3.42 ± 0.03 ^A^	3.24 ± 0.03 ^B^	0.10 ± 0.01 ^bE^	0.67 ± 0.04 ^bC^	0.14 ± 0.00 ^aD^	−0.01 ± 0.01 ^bF^	**
Sign	ns	ns	**	**	**	**	
b*							
C	6.38 ± 0.10 ^aA^	6.35 ± 0.13 ^aA^	2.11 ± 0.06 ^bD^	2.03 ± 0.05 ^bE^	2.95 ± 0.06 ^aB^	2.24 ± 0.02 ^bC^	**
MAVOO-M	5.93 ± 0.10 ^bA^	5.97 ± 0.03 ^bA^	3.34 ± 0.01 ^aC^	5.79 ± 0.03 ^aB^	2.48 ± 0.03 ^bD^	2.06 ± 0.07 ^cE^	**
MAVOO-I	6.38 ± 0.08 ^aA^	5.62 ± 0.02 ^cB^	2.05 ± 0.04 ^cE^	2.04 ± 0.02 ^bE^	2.97 ± 0.01 ^aC^	2.50 ± 0.03 ^aD^	**
Sign	**	**	**	**	**	**	
Chroma*							
C	7.23 ± 0.09 ^aA^	7.22 ± 0.11 ^A^	2.11 ± 0.06 ^bB^	2.15 ± 0.04 ^bB^	2.95 ± 0.06 ^cB^	2.24 ± 0.02 ^bB^	**
MAVOO-M	6.85 ± 0.09 ^bA^	7.34 ± 0.68 ^A^	6.72 ± 0.01 ^aA^	6.69 ± 0.04 ^aA^	2.49 ± 0.01 ^bB^	2.07 ± 0.07 ^cB^	**
MAVOO-I	7.21.0.05 ^aA^	6.49 ± 0.03 ^B^	2.05 ± 0.04 ^bE^	2.15 ± 0.02 ^bE^	2.98 ± 0.01 ^aC^	2.50 ± 0.03 ^aD^	**
Sign	**	ns	**	**	**	**	

Data are expressed as the mean ± standard deviation (*n* = 3). Statistical analysis was followed by Tukey’s test, which were used to evaluate any differences at the same time of analysis (lowercase letters) or during the considered storage (uppercase letters). Results followed by letters were significant at *p* ≤ 0.01. ** *p* ≤ 0.01; ns, not significant at *p* > 0.05.

**Table 3:** α-Amylase, α-glucosidase, and lipase inhibitory activities (IC50 μg/mL) of the unaromatized olive oil (C), aromatized olive oil by co-mixing 1% (MAVOO-M) and aromatized olive oil by infusion 2% (MAVOO-I).

Samples	Days of Storage						
	T0	T15	T30	T60	T180	T360	Sign
α-Amylase							
C	269.02 ± 3.77 ^aD^	275.21 ± 3.85 ^aCD^	303.38 ± 3.92 ^aB^	345.31 ± 4.05 ^aA^	240.29 ± 3.87 ^aE^	289.32 ± 4.90 ^bC^	**
MAVOO-M	189.40 ± 3.56 ^bE^	195.59 ± 3.77 ^bD^	200.44 ± 3.44 ^cD^	213.04 ± 3.35 ^cC^	229.52 ± 3.08 ^bB^	347.78 ± 3.50 ^aA^	**
MAVOO-I	189.47 ± 3.56 ^bD^	192.67 ± 3.81 ^bD^	208.72 ± 3.44 ^bC^	233.98 ± 3.35 ^bB^	237.01 ± 3.49 ^aB^	258.65 ± 3.8 ^cA^	**
Sign	**	**	**	**	**	**	
M	162.49 ± 3.26						
Acarbose	50.18 ± 1.32						
	α-Glucosidase						
C	137.34 ± 3.73 ^F^	145.18 ± 3.79 ^abE^	198.81 ± 3.82 ^aD^	337.56 ± 3.90 ^aC^	587.49 ± 3.56 ^aB^	778.23 ± 4.67 ^aA^	**
MAVOO-M	136.58 ± 3.45 ^E^	152.21 ± 3.47 ^aDE^	161.7 ± 3.79 ^cD^	183.23 ± 3.81 ^cC^	237.66 ± 3.88 ^cB^	344.87 ± 4.09 ^bA^	**
MAVOO-I	136.55 ± 3.45 ^E^	140.05 ± 3.81 ^bE^	172.18 ± 3.09 ^bD^	220.92 ± 3.01 ^bC^	267.89 ± 3.90 ^bB^	307.07 ± 4.21 ^cA^	**
Sign	ns	**	**	**	**	**	
M	206.17 ± 3.82						
Acarbose	35.57 ± 0.99						
	Lipase						
C	143.46 ± 4.85 ^aF^	155.52 ± 4.87 ^aE^	173.43 ± 4.91 ^aD^	206.54 ± 5.01 ^aC^	253.81 ± 4.81 ^aB^	312.97 ± 5.44 ^aA^	**
MAVOO-M	62.25 ± 1.09 ^bE^	67.20 ± 1.14 ^bDE^	70.54 ± 1.22 ^bD^	95.95 ± 1.72 ^bC^	119.32 ± 2.89 ^bB^	200.12 ± 3.05 ^bA^	**
MAVOO-I	62.33 ± 4.12 ^bE^	69.34 ± 4.22 ^bDE^	73.18 ± 4.22 ^bD^	94.99 ± 4.02 ^bC^	121.35 ± 4.87 ^bB^	138.66 ± 4.99 ^cA^	**
Sign	**	**	**	**	**	**	
M	83.60 ± 4.76						
Orlistat	37.44 ± 1.08						

Data are expressed as the mean ± standard deviation (*n* = 3). Statistical ANOVA was followed by Tukey’s test, which were used to evaluate any differences at the same time of analysis (lowercase letters) or during the considered storage (uppercase letters). Results followed by letters were significant at *p* ≤ 0.01. ** *p* ≤ 0.01; ns, not significant at *p* > 0.05.

## Data Availability

The raw data supporting the conclusions of this article will be made available by the authors upon request.
